# Fluorescence-guided assessment of bone and soft-tissue sarcomas for predicting the efficacy of telomerase-specific oncolytic adenovirus

**DOI:** 10.1371/journal.pone.0298292

**Published:** 2024-02-20

**Authors:** Koji Uotani, Hiroshi Tazawa, Joe Hasei, Tomohiro Fujiwara, Aki Yoshida, Yasuaki Yamakawa, Toshinori Omori, Kazuhisa Sugiu, Tadashi Komatsubara, Hiroya Kondo, Takuya Morita, Masahiro Kiyono, Suguru Yokoo, Toshiaki Hata, Toshiyuki Kunisada, Ken Takeda, Yasuo Urata, Toshiyoshi Fujiwara, Toshifumi Ozaki

**Affiliations:** 1 Department of Orthopaedic Surgery, Okayama University Graduate School of Medicine, Dentistry and Pharmaceutical Sciences, Okayama, Japan; 2 Center for Innovative Clinical Medicine, Okayama University Hospital, Okayama, Japan; 3 Department of Gastroenterological Surgery, Okayama University Graduate School of Medicine, Dentistry and Pharmaceutical Sciences, Okayama, Japan; 4 Department of Medical Materials for Musculoskeletal Reconstruction, Okayama University Graduate School of Medicine, Dentistry and Pharmaceutical Sciences, Okayama, Japan; 5 Oncolys BioPharma, Inc., Tokyo, Japan; Virginia Commonwealth University, UNITED STATES

## Abstract

Bone and soft-tissue sarcomas are rare malignancies with histological diversity and tumor heterogeneity, leading to the lack of a common molecular target. Telomerase is a key enzyme for keeping the telomere length and human telomerase reverse transcriptase (hTERT) expression is often activated in most human cancers, including bone and soft-tissue sarcomas. For targeting of telomerase-positive tumor cells, we developed OBP-301, a telomerase-specific replication-competent oncolytic adenovirus, in which the hTERT promoter regulates adenoviral *E1* gene for tumor-specific viral replication. In this study, we present the diagnostic potential of green fluorescent protein (GFP)-expressing oncolytic adenovirus OBP-401 for assessing virotherapy sensitivity using bone and soft-tissue sarcomas. OBP-401-mediated GFP expression was significantly associated with the therapeutic efficacy of OBP-401 in human bone and soft-tissue sarcomas. In the tumor specimens from 68 patients, malignant and intermediate tumors demonstrated significantly higher expression levels of coxsackie and adenovirus receptor (CAR) and hTERT than benign tumors. OBP-401-mediated GFP expression was significantly increased in malignant and intermediate tumors with high expression levels of CAR and hTERT between 24 and 48 h after infection. Our results suggest that the OBP-401-based GFP expression system is a useful tool for predicting the therapeutic efficacy of oncolytic virotherapy on bone and soft-tissue sarcomas.

## Introduction

Bone and soft-tissue sarcomas are a group of rare malignant tumors of mesenchymal origin showing histological diversity [1,2] and tumor heterogeneity [3–5]. Bone and soft-tissue sarcomas have more than 80 histological subtypes [6]. Although precision medicine based on comprehensive genomic profiling has been developed for patients with cancer with druggable target molecules [7], the frequency of druggable genetic alterations in bone and soft-tissue sarcomas has been only 5% [8]. Of note, genetic, phenotypic, and microenvironmental heterogeneity plays a critical role in antitumor modality resistance [9]. Histological diversity and tumor heterogeneity lead to the lack of common molecular targets for treating patients with bone and soft-tissue sarcomas. Therefore, to develop novel therapeutic options for efficiently eliminating bone and soft-tissue sarcoma cells, a commonly activated molecular target is needed.

Oncolytic virotherapy became a novel antitumor therapy for inducing tumor-specific cell lysis based on commonly activated molecular targets in tumor cells [10]. Telomerase is a key enzyme for keeping the telomere length, and human telomerase reverse-transcriptase (hTERT) expression is frequently activated in most human cancers including bone and soft-tissue sarcomas [11,12]. To target telomerase-positive tumor cells, we developed a telomerase-specific replication-competent oncolytic adenovirus OBP-301, in which the hTERT promoter regulates adenoviral the *E1* gene for tumor-specific viral replication. OBP-301 induces antitumor activity in various human cancer cells, including bone and soft-tissue sarcomas, by binding to the coxsackie and adenovirus receptor (CAR) on their surface [13–15]. OBP-301 also enhances the efficacies of radiotherapy and chemotherapy against human bone and soft-tissue sarcomas [16–18]. In a phase I clinical study of OBP-301 conducted in the United States on patients with advanced solid tumors including sarcoma, patients well tolerated OBP-301 [19]. For the clinical application of OBP-301, a predictive biomarker is needed for selecting patients with cancer who will benefit from OBP-301-based oncolytic virotherapy.

The therapeutic efficacy of OBP-301 mainly depends on the infectivity and replication capability against target tumor cells. Therefore, CAR and hTERT expressions in tumor cells must be evaluated. Immunohistochemistry is often used as a conventional method for evaluating the expression levels of CAR and hTERT in tumor tissues [20,21]. Thus, to evaluate the therapeutic efficacy of OBP-301 in tumor cells more simply than immunohistochemistry, we developed a green fluorescent protein (GFP)-expressing OBP-301 variant (OBP-401) [22]. OBP-401 induces GFP expression in human bone and soft-tissue sarcoma cells with telomerase activity and CAR expression, but not in normal cells [23]. OBP-401 and OBP-301 have cytopathic effects and is against cancer cells in human [24]. Moreover, OBP-401 induces GFP expression in malignant tumor cells within normal tissues for in vivo imaging of lymph node metastasis [22,25], liver metastasis [26], peritoneal metastasis [27], and fluorescence-guided surgery [28]. Therefore, we hypothesized that the OBP-401-mediated GFP expression system is a useful tool for predicting the therapeutic efficacy of OBP-301 by using clinical tumor specimens of bone and soft-tissue sarcomas.

In this study, we evaluated the relationship between the expression levels of CAR and hTERT and OBP-401-mediated GFP expression and cytopathic activity in human bone and soft-tissue sarcoma cells and normal fibroblasts. Moreover, for the clinical application of oncolytic virotherapy to bone and soft-tissue sarcomas, we analyzed clinical tumor specimens from 68 patients to evaluate the relationship between the expression levels of CAR and hTERT and OBP-401-mediated GFP expression.

## Materials and methods

### Cell lines

The human osteosarcoma cell lines HOS (CRL-1543), MNNG/HOS (CRL-1547), and 143B (CRL-8303) were purchased from the American Type Culture Collection (ATCC, Manassas, VA, USA). The human synovial sarcoma cell line SYO-1 was previously established in our laboratory [29]. Normal skeletal muscle tissues were cultured on a collagen-coated dish containing a culture medium that promotes fibroblast growth. After 1 week of culture, normal fibroblasts have grown. Subsequently, primary cultures of normal fibroblasts were established, as reported previously [30]. Sarcoma cells and normal fibroblasts were maintained in Dulbecco’s modified Eagle’s medium (Gibco Laboratories, Grand Island, NY, USA) supplemented with 10% fetal bovine serum (HyClone, Logan, UT, USA), 100 units/mL penicillin G, and 100 μg/mL streptomycin (Nacalai Tesque, Inc., Kyoto, Japan). Cells were incubated at 37°C in a humidified atmosphere containing 5% CO_2_.

### Recombinant adenovirus

OBP-401 was obtained from Oncolys BioPharma, Inc. (Tokyo, Japan). OBP-401 is a GFP-expressing variant of telomerase-specific replication-competent oncolytic adenovirus OBP-301, in which the *hTERT* promoter activates the expressions of *E1A* and *E1B* genes associated with an internal ribosome entry site for tumor-specific viral replication. The *GFP* gene expression cassette under the control of cytomegalovirus promoter was inserted into the *E3* region of OBP-301 [22]. Their titers were determined by a plaque-forming assay using 293 cells, and they were stored at –80°C.

### Flow cytometric analysis

To analyze CAR expression, 10^6^ cells in each sample were incubated with PE-conjugated rabbit anti-CAR monoclonal antibody (271; Thermo Fisher Scientific, Waltham, MA, USA) or PE-conjugated rabbit isotype IgG (406421; Biolegend, San Diego, CA, USA) for 30 min at 4°C, and 10^4^ events in each sample were recorded and then analyzed using MACSQuant (Miltenyi Biotec GmbH, Germany) at the Central Research Laboratory, Okayama University Medical School. In triplicate experiments, the mean fluorescence intensity (MFI) for each cell line was determined by calculating the difference between the MFI in antibody-incubated and isotype IgG-incubated cells.

### Real-time quantitative reverse-transcription polymerase chain reaction (RT-qPCR)

Total RNA from cells was obtained using an RNeasy Mini Kit (Qiagen, Valencia, CA, USA) according to the manufacturer’s instructions. The *hTERT* mRNA expression was examined by RT-qPCR. Reverse transcription was carried out using the PrimerScript RT Reagent Kit (Takara Bio, Tokyo, Japan) according to the manufacturer’s protocol. RT-qPCR was performed using the Quantstudio1 (Thermo Fisher Scientific) with TaqMan 2 × Universal PCR Master Mix (Thermo Fisher Scientific) and TaqMan probes for human (glyceraldehyde-3-phosphate dehydrogenase (GAPDH); Hs02786624_g1 and hTERT; Hs00972656_m1, Thermo Fisher Scientific). Data obtained from the RT-qPCR were analyzed using the 2^-ΔΔCt^ method after normalization with reference to the expression of *GAPDH* mRNA. The values of *hTERT* mRNA expression in normal fibroblasts were set at 1, and relative levels of *hTERT* mRNA expression in each cell were plotted as fold expression.

### OBP-401 infection protocol using malignant tumor and normal cells

Cells were seeded into 96-well plates at 10^3^ cells per well 24 h before infection. Then, the cells were infected with OBP-401 at a multiplicity of infection (MOI) of 0, 1, 5, 10, 50, or 100 plaque-forming units (PFU)/cell at 37°C in a humidified atmosphere containing 5% CO_2_. The expression of GFP was evaluated 0, 24, 48, or 72 h after OBP-401 infection using FlexStation3 (Molecular Devices, Sunnyvale, CA, USA).

### WST-1 assay

Before treatment, cells were seeded in 96-well plates at a density of 10^3^ cells/well 24 h. Cells were infected with OBP-401 at an MOI of 0, 1, 5, 10, 50, or 100 PFU/cell for 72 h. Cell viability was determined 24, 48, or 72 h after OBP-401 infection using the WST-1 assay (Roche Molecular Biochemicals, Mannheim, Germany) according to the manufacturer’s protocols. iMark Microplate Absorbance Reader (Bio-Rad, Hercules, CA, USA) was used to evaluate absorbance at the indicated times.

### Clinical tumor specimens

This study protocol was approved by the Institutional Review Board of Okayama University Hospital (No. 1854). After study approval, written informed consent was obtained from all patients. All experimental methods were conducted in accordance with relevant guidelines and regulations. The tumor specimens of the primary bone and soft-tissue tumor were obtained from 68 patients who underwent surgery at Okayama University Hospital between January 2015 and July 2016. Table 1 presents the pathological diagnoses of primary bone and soft-tissue tumors classified into malignant (n = 48), intermediate (n = 6), and benign (n = 14) groups.

**Table 1:** Pathological diagnosis of clinical tumor specimens.

Malignant group	n	Intermediate group	n	Benign group	n
Bone tumor		Bone tumor		Bone tumor	
Osteosarcoma	4	Giant cell tumor	2	Chondroblastoma	1
Chondrosarcoma	4				
Soft-tissue tumor		Soft-tissue tumor		Soft-tissue tumor	
Undifferentiated pleomorphic sarcoma	9	Dermatofibrosarcoma protuberans	2	Schwannoma	9
Myxofibrosarcoma	8	Solitary fibrous tumor	2	Tenosynovial giant cell tumor	3
Dedifferentiated liposarcoma	6			Lipoma	1
Malignant peripheral nerve sheath tumor	6				
Leiomyosarcoma	4				
Synovial sarcoma	2				
Myxoid liposarcoma	2				
Alveolar soft part tissue sarcoma	1				
Pleomorphic liposarcoma	1				
Clear cell sarcoma	1				
Total	48	Total	6	Total	14

### Immunohistochemistry

Tissue sections (4 μm) were deparaffinized, rehydrated with serial reduced concentration of ethanol, and stained with rabbit anti-CAR polyclonal antibody (CAR H300; Santa Cruz Biotechnology, Santa Cruz, CA, USA) or anti-hTERT polyclonal antibody (ab183105; Abcam, Cambridge, UK) at 4°C overnight, followed by incubation with the second antibody (Histfine Simple Stain MAX-PO (Multi) Kit; Nichirei, Tokyo, Japan) for 30 min at room temperature and detected with an ABC complex, followed by diaminobenzidine and hematoxylin counterstaining. All tissue samples were assessed by the averaged threshold method (ATM), as reported previously [31]. According to the same threshold for each sample, the staining intensity and percent area stained were assessed using cellSence software (Olympus, Tokyo, Japan). Then, the intensity was multiplied by the percent for each area stained, and the total of each result was used as the ATM score.

### OBP-401 infection protocol using clinical tumor specimens

Tumor specimens resected from patients were immediately mercerized into 2 × 2 × 2 mm^3^ under a sterile condition. These samples were divided into the OBP-401 infection group and the control PBS group (n = 3 each) on 96-well plates with a culture medium. As a single tumor cell is approximately 10 μm in diameter, the number of tumor cells on the surface area of each tissue specimen (2 × 2 × 2 mm^3^) was estimated as 2.4 × 10^5^ cells. Then, 2.4 × 10^6^ PFU of OBP-401 for each tissue specimen (2 × 2 × 2 mm^3^) indicates approximately 10 MOI for each tumor tissue. In the OBP-401 infection group, 2.4 × 10^6^ PFU of OBP-401 was administered immediately after trimming tumor specimens, as reported previously [23]. The extended focus imaging of the GFP expression emitted from the OBP-401-infected tumor samples was captured by an inverted fluorescence microscope IX-73 (Olympus, Tokyo, Japan). In triplicate, GFP expression was evaluated at the indicated time by estimating the percentage of the area where the GFP expression was positive to the entire field of tissue specimens.

### Statistical analysis

Values are presented as the mean ± standard deviation or the median with a 25%−75% range. Statistical differences in the MFI of CAR and the expression of hTERT mRNA between sarcoma cells and normal fibroblasts were calculated using an unpaired t-test. Statistical differences in the evaluation of the relevance between GFP intensity and cell viability by the WST-1 assay were computed by nonparametric Spearman correlation. Statistical differences in GFP intensity and ATM scores of CAR and hTERT between the malignant and benign groups were calculated using an unpaired t-test with Welch’s correction. A two-sided *p*-value of <0.05 was considered statistically significant. Statistical analyses were conducted using GraphPad Prism version 6.0 h (GraphPad Software, San Diego, CA, USA).

## Results

### CAR and hTERT expressions in human bone and soft-tissue sarcoma cells

**Fig 1:**
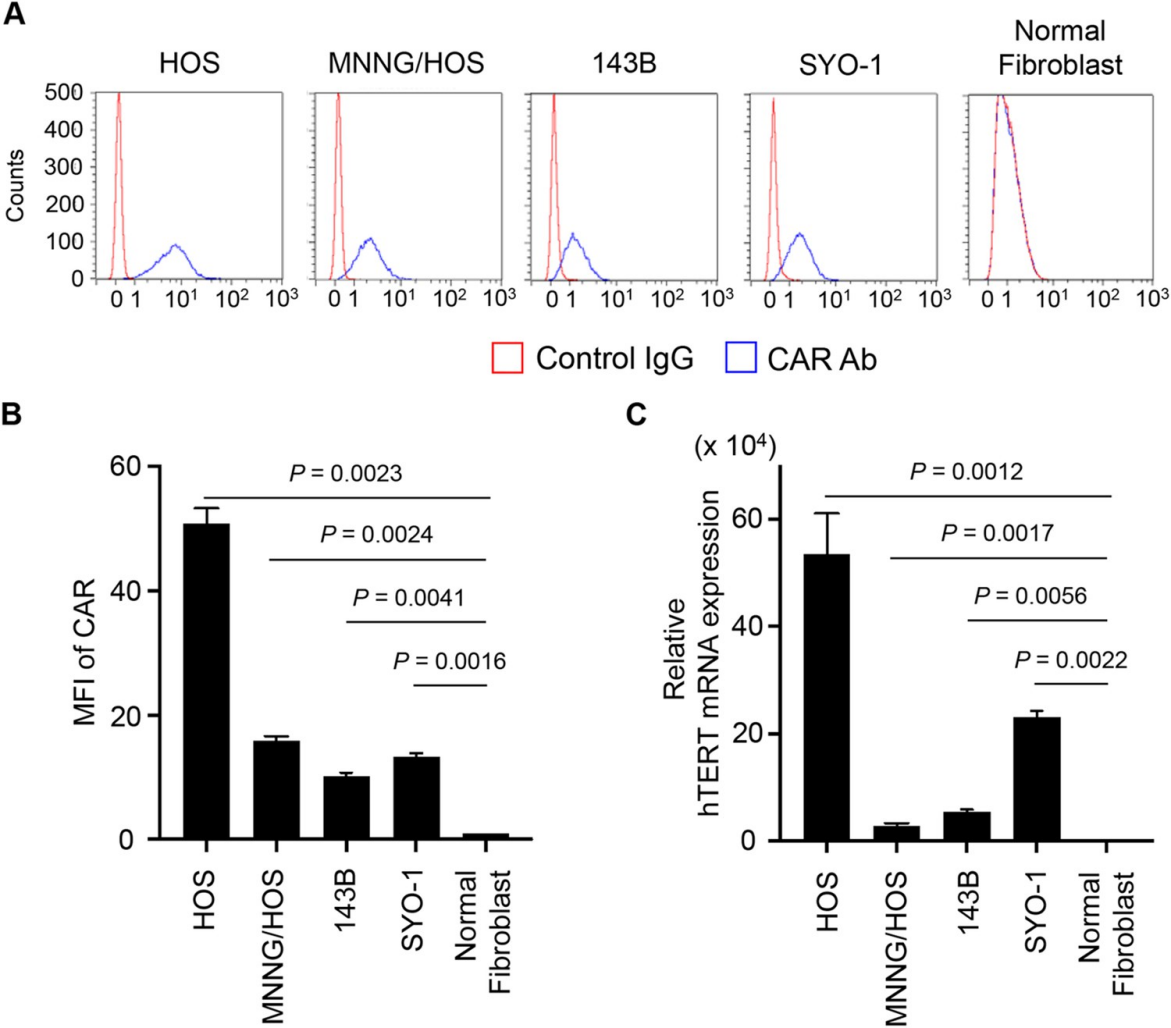
CAR and hTERT expression levels in human bone and soft-tissue sarcoma cells. (**A**,**B**) The CAR expression on the surface of bone and soft-tissue sarcoma cells and fibroblasts was analyzed by flow cytometric analysis. Representative histograms in each sample were shown (**A**). The mean fluorescence intensity (MFI) for each cell was determined by calculating the differences between the MFI in antibody-incubated and isotype IgG-incubated cells (**B**). (**C**) The hTERT mRNA expression in bone and soft-tissue sarcoma cells and fibroblasts was analyzed by RT-PCR. Data are expressed as mean values ± SD of independent experiments (n = 3). The statistical significance of the differences was calculated using an unpaired t-test. N.S., not significant.

To evaluate the expression levels of CAR and hTERT in bone and soft-tissue sarcoma cells, we used isogenic human osteosarcoma cells with different malignant potential (i.e., parental HOS cells without tumorigenic potential, MNNG/HOS cells with tumorigenic potential, and 143B cells with tumorigenic and metastatic potentials), SYO-1 cells derived from human synovial sarcoma (soft-tissue sarcoma), and human NFs. The expression level of CAR on the surface of sarcoma cells and normal fibroblasts were analyzed by flow cytometry. HOS cells demonstrated higher levels of CAR expression than MNNG/HOS, 143B, and SYO-1 cells, whereas normal fibroblasts had low CAR expression (Fig 1A and 1B). There were significant differences in the CAR expression between sarcoma cells and normal fibroblasts (Fig 1B). The expression of hTERT mRNA in sarcoma cells and normal fibroblasts was analyzed by RT-PCR. HOS cells exhibited higher levels of *hTERT* mRNA expression than MNNG/HOS, 143B, and SYO-1 cells, whereas normal fibroblasts showed low expression (Fig 1C). There were significant differences in the hTERT expression between sarcoma cells and normal fibroblasts (Fig 1C). These results suggest that bone and soft-tissue sarcoma cells have varied expressions of CAR and hTERT, whereas normal fibroblasts possess low expressions of CAR and hTERT.

### Relationship between OBP-401-mediated GFP expression and cytopathic activity in human bone and soft-tissue sarcoma cells

**Fig 2:**
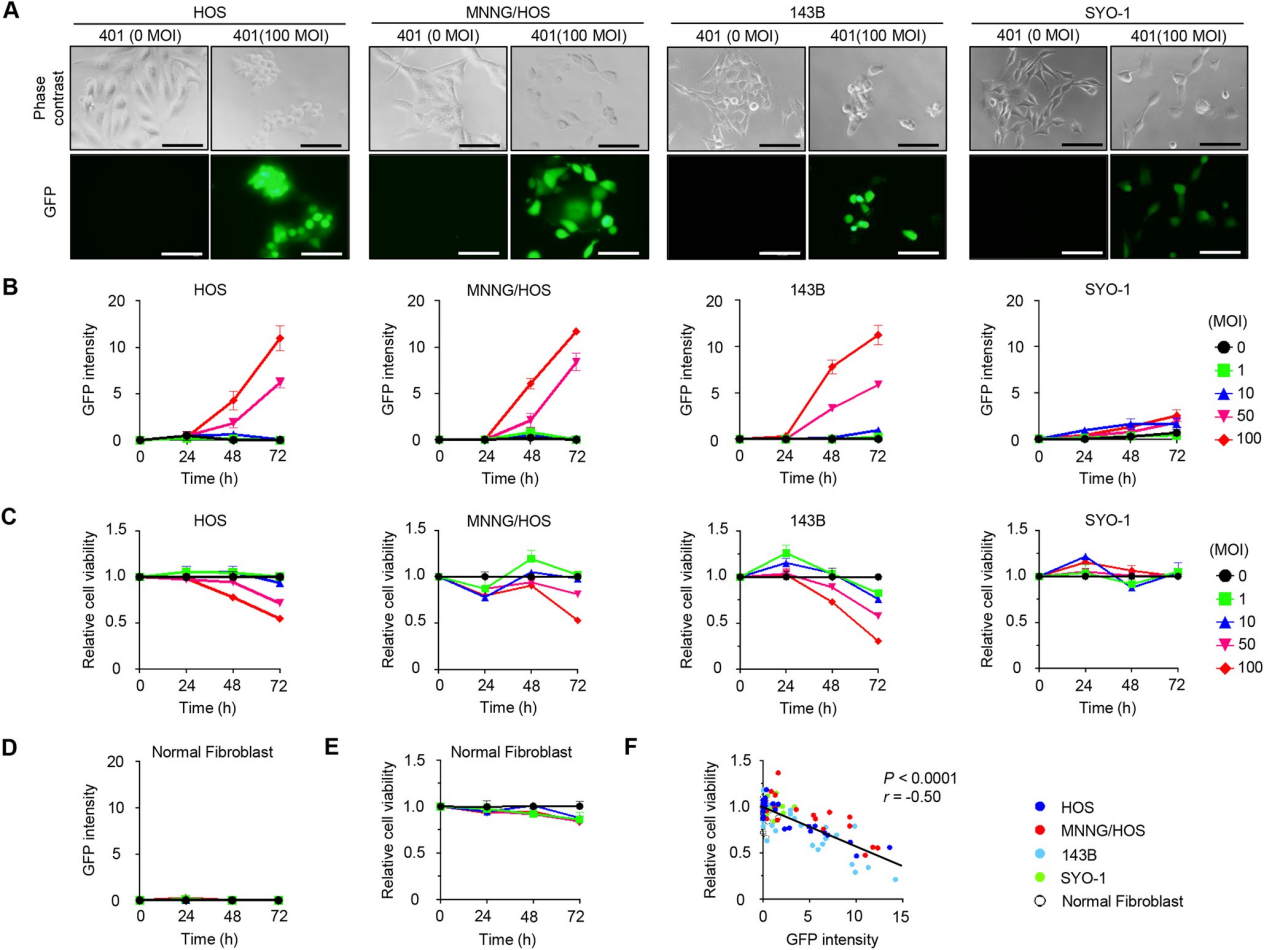
OBP-401-mediated GFP expression and cell viability in human bone and soft-tissue sarcoma cells. (**A**) Representative photographs of bone and soft-tissue sarcoma cells 72 h after infection with OBP-401 at the indicated doses. Scale bars: 100 μm. (**B**,**D**) The GFP intensity was analyzed using FlexScan in bone and soft-tissue sarcoma cells (**B**) and normal fibroblasts (**D**) 24, 48, and 72 h after infection with OBP-401 at the indicated doses. Data are expressed as mean values ± SD of independent experiments (n = 3). (**C**,**E**) Cell viability was analyzed using the WST-1 assay in bone and soft-tissue sarcoma cells (**C**) and normal fibroblasts (**E**) at 24, 48, and 72 h after infection with OBP-401 at the indicated doses. Data are expressed as mean values ± SD of independent experiments (n = 5). (**F**) Relationship between the GFP intensity and cell viability in bone and soft-tissue sarcoma cells and fibroblasts infected with OBP-401.

To investigate the relationship between OBP-401-mediated GFP expression and cytopathic activity in bone and soft-tissue sarcoma cells, GFP expression was analyzed in sarcoma cells and fibroblasts after infection with OBP-401 at an MOI of 0, 1, 5, 10, 50, or 100 PFU/cell for 24, 48, and 72 h under a fluorescence microscope. When infected with OBP-401 at high doses (50 and 100 MOIs), HOS, MNNG/HOS, and 143B cells demonstrated time-dependent increased GFP expressions, and SYO-1 cells showed a slight increase in GFP expression (Fig 2A and 2B). Then, the cell viability was assessed in sarcoma cells and fibroblasts after OBP-401 infection for 24, 48, and 72 h using the WST-1 assay. OBP-401 infection at high doses (50 and 100 MOIs) significantly decreased the viability of HOS, MNNG/HOS, and 143B cells, whereas SYO-1 cells were resistant to OBP-401 (Fig 2C). By contrast, GFP expression was undetectable in normal fibroblasts and these cells were resistant to OBP-401 (Fig 2D and 2E). A significantly inverse correlation was found between GFP expression and cell viability in the OBP-401-infected malignant tumor and normal cells (*P* < 0.0001, *r* = −0.50) (Fig 2F). Thus, OBP-401-mediated GFP expression is a promising biomarker for predicting the virus-mediated cytopathic efficacy in human bone and soft-tissue sarcoma cells.

### CAR and hTERT expressions in clinical tumor specimens of bone and soft-tissue tumors

**Fig 3:**
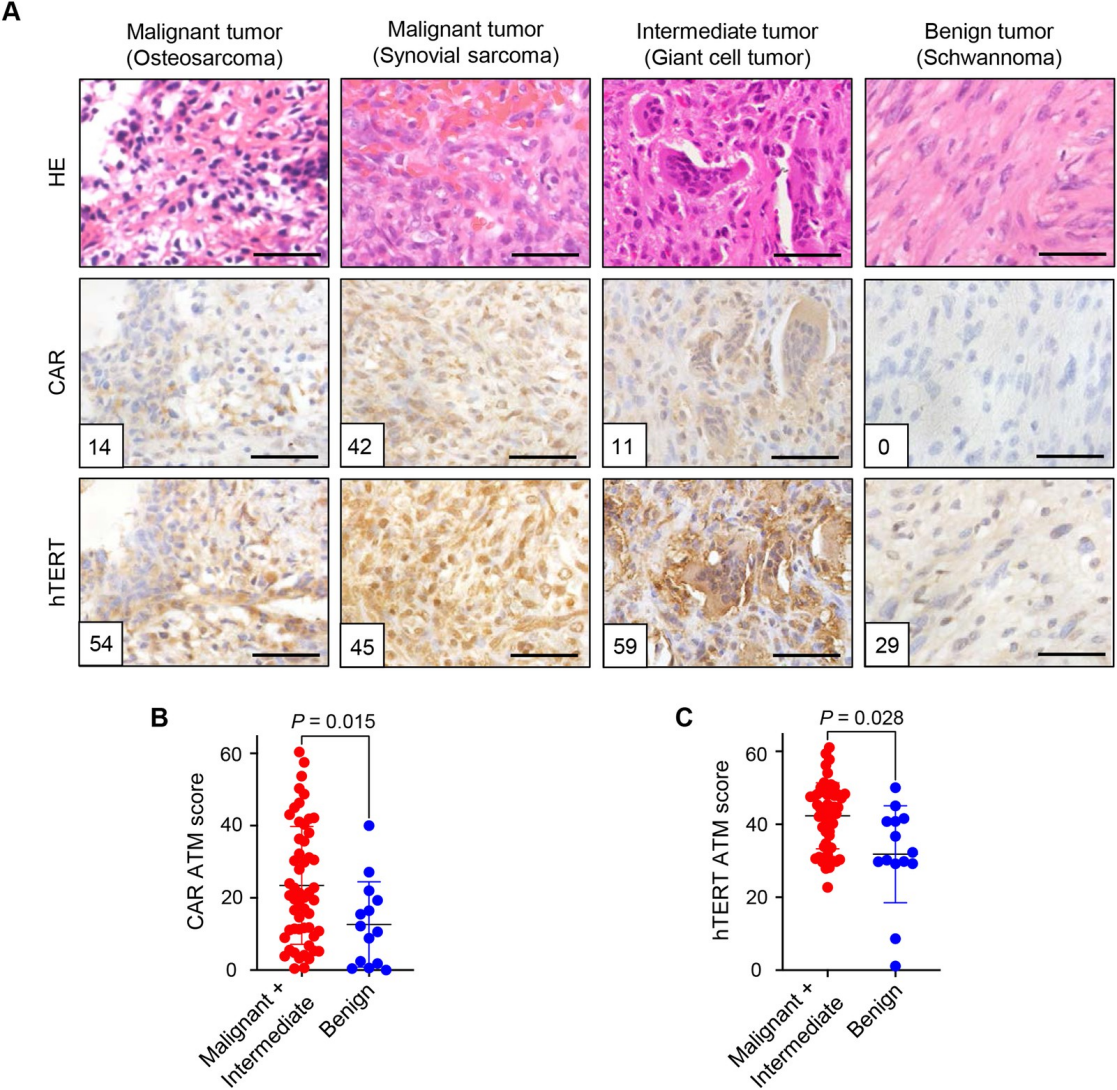
CAR and hTERT expressions in clinical specimens of bone and soft-tissue tumors. (**A**) Representative photographs of immunohistochemical staining for CAR and hTERT using clinical tumor specimens. The top panel shows hematoxylin and eosin (HE) staining, the middle panel shows CAR expression, and the bottom panel shows hTERT expression. The ATM score is shown in each photograph. Scale bars: 50 μm. (**B**,**C**) Comparison of ATM score for CAR (**B**) or hTERT (**C**) between the malignant (n = 48) and intermediate (n = 6) group and the benign (n = 14) group. The statistical significance was determined using the Mann−Whitney test.

To evaluate the expression levels of CAR and hTERT in bone and soft-tissue sarcoma tissues, clinical tumor specimens from 68 patients were analyzed by immunohistochemistry. Tumor samples with malignant (n = 48), intermediate (n = 6), and benign (n = 14) potential included various histological subtypes of bone and soft-tissue tumors (Tables 1 and S1). Since intermediate tumors show clinically aggressive behavior similar to malignant tumors, we divided the tumor samples into the malignant and intermediate group and the benign group. In the malignant and intermediate group including osteosarcomas, synovial sarcomas, and giant cell tumors, CAR expression was observed in the cytoplasm and membrane of tumor cells, whereas hTERT expression was observed in the nuclear and cytoplasm of tumor cells (Fig 3A). In the benign group including schwannoma, low or undetectable expressions of CAR and hTERT were observed in tumor cells (Fig 3A). The expression levels of CAR and hTERT in the malignant and intermediate group and the benign group were evaluated using the ATM, as reported previously [31]. The ATM score of CAR expression was significantly higher in the malignant and intermediate group than in the benign group (*P* = 0.015) (Fig 3B). Moreover, the malignant and intermediate group showed significantly higher hTERT expression than the benign group (*P* = 0.028) (Fig 3B). These results suggest that bone and soft-tissue tumors with malignant and intermediate potentials exhibit high expression levels of CAR and hTERT.

### OBP-401-mediated GFP expression in clinical tumor specimens of bone and Soft-tissue tumors

**Fig 4:**
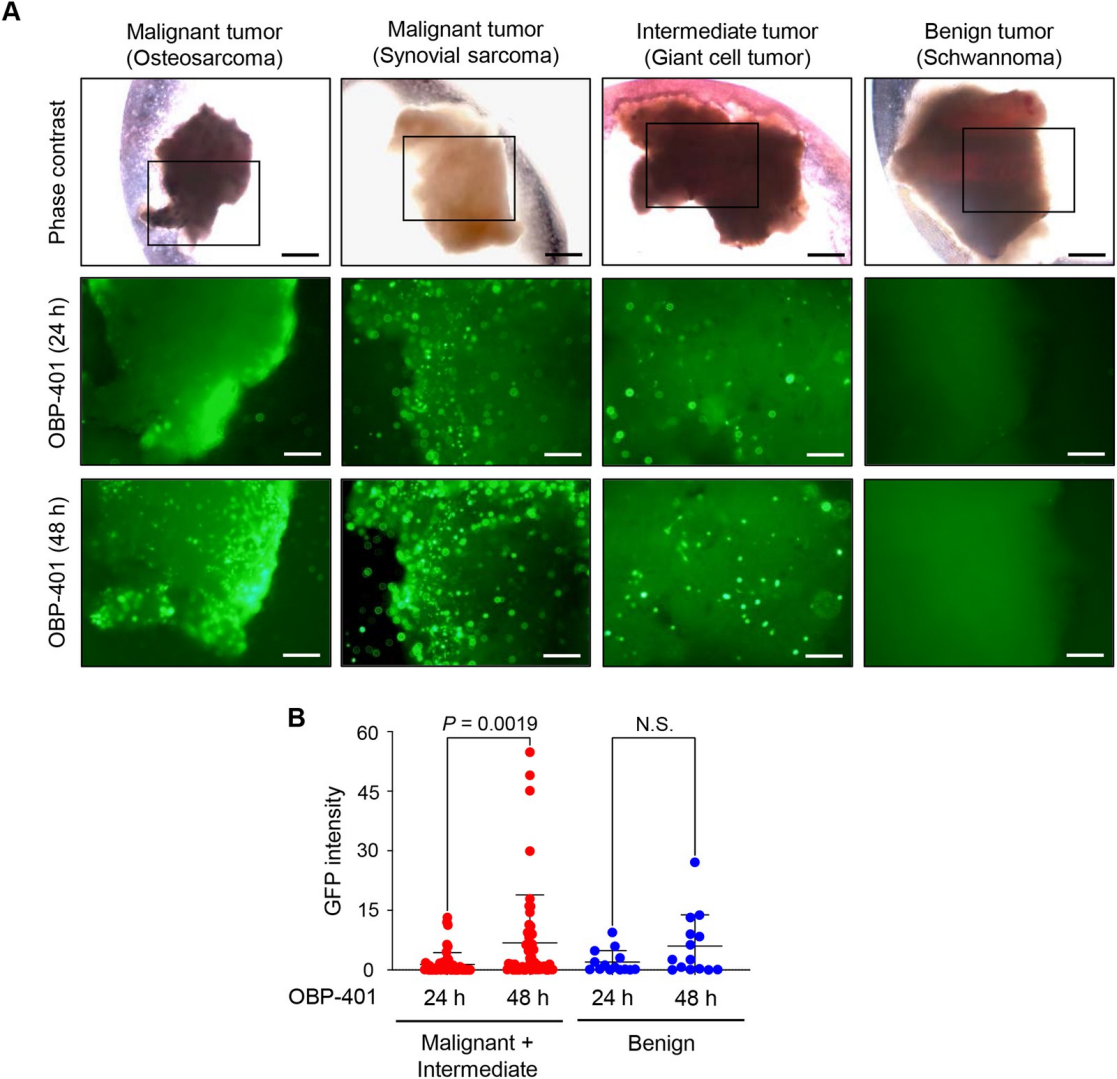
OBP-401-mediated GFP expression in clinical specimens of bone and soft-tissue tumors. (**A**) Representative photographs of tumor specimens at 24 and 48 h after OBP-401 infection. The top panel shows phase-contrast images, and the bottom panel shows fluorescent images for GFP expression. The scale bars represent 1 mm and 500 μm in the top and bottom panels, respectively. (**B**) The GFP intensity was compared between 24 and 48 h after OBP-401 infection in the malignant (n = 48) and intermediate (n = 6) group and the benign (n = 14) group. The statistical significance of the differences was calculated using an unpaired t-test. N.S., not significant.

To evaluate whether OBP-401 induces GFP expression in bone and soft-tissue tumors, clinical tumor specimens with malignant (n = 48), intermediate (n = 6), and benign (n = 14) potential were infected with OBP-401 for 48 h (S1 Table). GFP expression in the tumor tissues 24 and 48 h after OBP-401 infection was evaluated under a fluorescence microscope. In the malignant and intermediate group, heterogeneous GFP expression was observed in tumor tissues (Fig 4A). In the benign group, very low or undetectable GFP expression was observed in tumor tissues (Fig 4A). The malignant and intermediate group had significantly increased GFP expression between 24 and 48 h after OBP-401 infection (*P* = 0.0019) (Fig 4B). However, the benign group showed no significant difference in GFP expression between 24 and 48 h after OBP-401 infection (Fig 4B). Thus, OBP-401 has a potential to induce GFP expression in malignant- and intermediate-grade bone and soft-tissue tumors.

### Relationship between CAR and hTERT expressions and OBP-401-mediated GFP expression in clinical tumor specimens of bone and soft-tissue tumors

**Fig 5:**
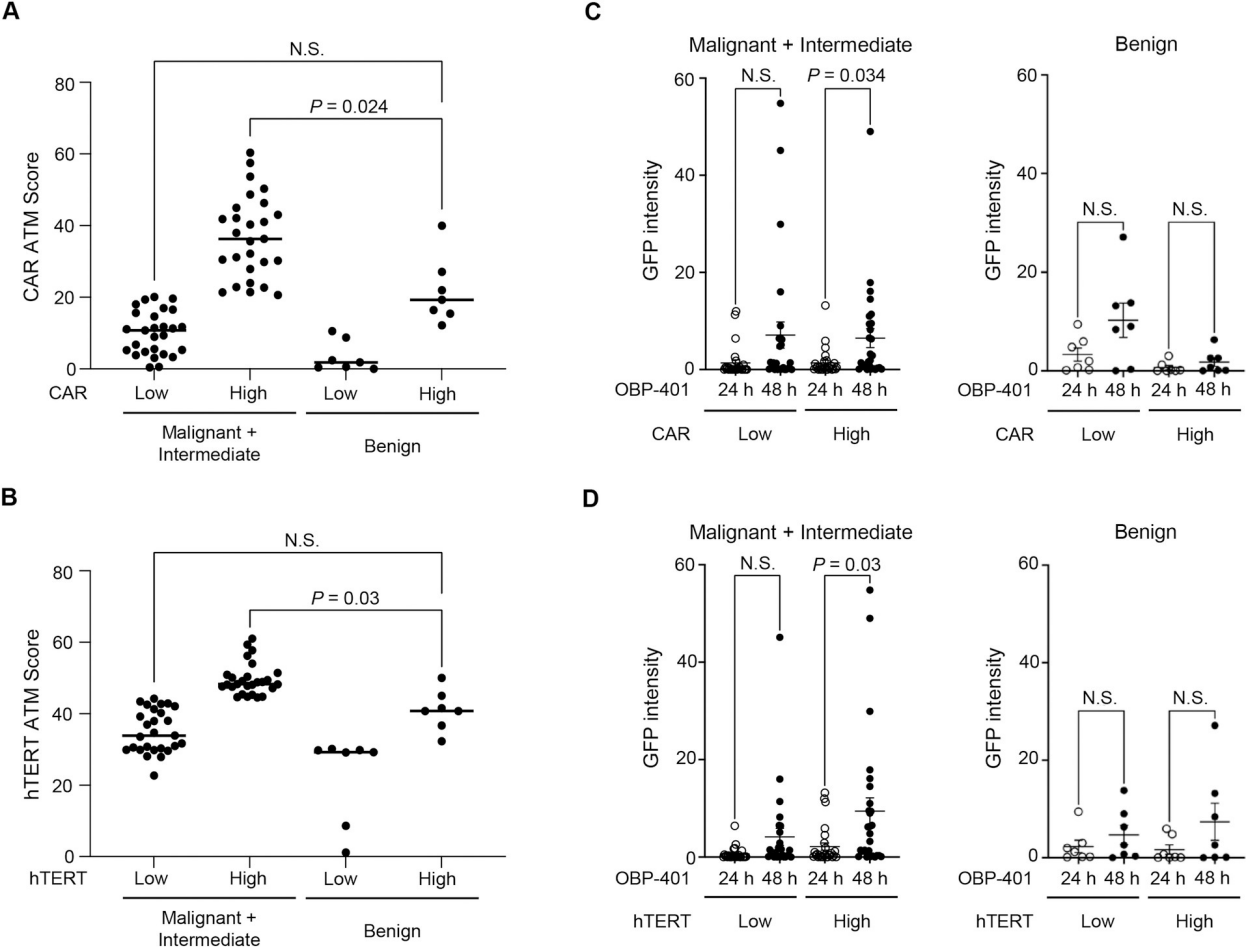
OBP-401-mediated GFP expression in clinical specimens of bone and soft-tissue tumors with different expressions of CAR and hTERT. (**A**,**B**) Comparison of ATM score for CAR (**A**) or hTERT (**B**) between the high and low group in the malignant (n = 48) and intermediate (n = 6) group and the benign (n = 14) group. (**C,D**) The GFP intensity was compared between 24 and 48 h after OBP-401 infection in the malignant and intermediate group and the benign group with different expressions of CAR (**C**) and hTERT (**D**). The statistical significance of the differences was calculated using an unpaired t-test. N.S., not significant.

Finally, to evaluate the relationship between the expression levels of CAR and hTERT and OBP-401-mediated GFP expression in bone and soft-tissue tumors, we subtyped 68 soft-tissue tumors with malignant (n = 48), intermediate (n = 6), and benign (n = 14) potential into CAR low/high and hTERT low/high groups (S1 Table). The high type of benign group showed almost similar expression levels of CAR and hTERT with the low type of intermediate and malignant group (Fig 5A and 5B). OBP-401-mediated GFP expression was compared between clinical tumor specimens with different expressions of CAR and hTERT. In the malignant and intermediate group, tumors with high CAR expression exhibited significant increase in GFP expression between 24 and 48 h after OBP-401 infection (*P* = 0.034) (Fig 5C). Tumors with high hTERT expression also showed a significant increase in GFP expression (*P* = 0.03) (Fig 5D). However, malignant and intermediate tumors with low CAR or hTERT expression showed no significant increase in GFP expression (Fig 5C and 5D). By contrast, benign tumors showed no significant increase in GFP expression between 24 and 48 h after OBP-401 infection regardless of CAR and hTERT expressions (Fig 5C and 5D). Thus, the OBP-401-mediated GFP expression system is a useful method in identifying bone and soft-tissue tumors with high CAR and hTERT expressions.

## Discussion

Oncolytic virotherapy became a novel antitumor therapy for inducing tumor-specific cell lysis. However, the predictive biomarker for assessing the therapeutic efficacy of oncolytic viruses is not yet established. Thus, by using human bone and soft-tissue sarcoma cells and clinical tumor specimens, we evaluated the relationship between the expression levels of CAR and hTERT and OBP-401-mediated GFP expression and cytopathic activity. In in vitro experiments, a significant correlation was found between OBP-401-mediated GFP expression and cytopathic activity against human bone and soft-tissue sarcoma cells. In ex vivo experiments using clinical tumor specimens, the expression levels of CAR and hTERT were significantly higher in the malignant and intermediate group than in the benign group. In the malignant and intermediate group, GFP expression was significantly increased in tumors with high CAR and hTERT expressions between 24 and 48 h after OBP-401 infection. Our results suggest that the OBP-401-mediated GFP expression system is a useful method for assessing virotherapy sensitivity in bone and soft-tissue tumors.

Non-tumorigenic HOS cells showed higher CAR and hTERT expressions than tumorigenic MNNG/HOS and metastatic 143B cells (Fig 1), suggesting the dynamic changes in CAR and hTERT expressions during tumor progression. Despite the different expression levels of CAR and hTERT, these osteosarcoma cells showed similar sensitivity to OBP-401-mediated GFP expression and cytopathic activity (Fig 2).

OBP-301 had a broad-spectrum cytopathic activity against human bone and soft-tissue sarcoma cells with different expressions of CAR and hTERT [15]. Therefore, OBP-401-mediated GFP expression may be more suitable biomarker than CAR and hTERT expression for assessing virotherapy sensitivity in malignant tumor cells.

In the malignant and intermediate group, bone and soft-tissue tumors showed higher CAR expression than those in the benign group (Fig 3). CAR expression is frequently upregulated in bone and soft-tissue sarcomas at mRNA and protein levels [32,33]. Gu et al. revealed that CAR mRNA expression was upregulated in 15 (75%) of 20 OS tumors [33]. Many bone and soft-tissue sarcomas showed high CAR mRNA expression, which varied in malignant tumors with the same histology [33]. By contrast, Kuster et al. showed that hypoxic environmental factors reduce CAR expression in human cancer cells [34]. CAR expression in tumor cells was reported to be upregulated or downregulated during tumor progression [35]. Thus, heterogeneous CAR expression in tumor cells may contribute to the different sensitivity to oncolytic adenoviruses in bone and soft-tissue sarcoma tissues.

hTERT expression was significantly increased in bone and soft-tissue tumors in the malignant and intermediate group compared with the benign group (Fig 3), consisting of various malignant tumors [36]. In bone and soft-tissue tumors, telomerase activity was positive in 81% of musculoskeletal sarcomas and 14% of benign tumors using clinical tumor samples [12]. Moreover, hTERT expression was positive in 46% of bone and soft-tissue sarcomas and 13% of benign bone and soft-tissue tumors [37]. These findings indicate the expression of hTERT in some benign tumors. In this study, hTERT expression was high in some benign tumors (Fig 3). However, OBP-401-mediated GFP expression was not significantly increased in the benign group (Fig 4). The high type of benign group showed almost similar expression levels of CAR with the low type of intermediate and malignant group (Fig 5). As malignant and intermediate tumors with low CAR expression showed no significant increase in GFP expression, benign tumors with high hTERT expression may less respond to OBP-401-mediated GFP expression due to low CAR expression.

The expression levels of CAR and hTERT in bone and soft-tissue sarcoma cells can affect the cytopathic efficacy of OBP-301 [15]. Therefore, CAR and hTERT expressions must be evaluated for the clinical application of OBP-301 to bone and soft-tissue sarcomas. A significant correlation was found between the OBP-401-induced GFP expression and the expression levels of CAR and hTERT in bone and soft-tissue sarcomas with malignant and intermediate potentials (Fig 5). Notably, high CAR and hTERT expressions were significantly associated with increased GFP expression between 24 and 48 h after OBP-401 infection (Fig 5). These results suggest that appropriate patients who benefit from OBP-301 treatment can be identified by applying OBP-401 to tumor specimens obtained from biopsy or operative surgery and by evaluating the GFP expression. However, whether OBP-401 induces the cytopathic effect against tumor specimens following GFP induction remains unclear. Further experiments are needed to evaluate whether OBP-401-mediated GFP expression is associated with cytopathic effect in clinical tumor samples. As a patient-derived xenograft (PDX) tumor model is useful for the evaluation of the therapeutic potential of OBP-301 against human oral cancer tumors [38], further experiments using a PDX model with human bone and soft-tissue sarcomas are warranted to evaluate the relationship between the OBP-401-mediated GFP expression and therapeutic efficacy of OBP-301 in bone and soft-tissue sarcoma tissues.

In conclusion, the results of this study demonstrated that the assessment of GFP expression after OBP-401 infection is a reliable alternative method of evaluating virotherapy sensitivity in association with CAR and hTERT expressions using malignant- and intermediate-grade bone and soft-tissue tumors with malignant and intermediate potential. This method would support the selection of patients with bone and soft-tissue tumors eligible for treatment with OBP-301, providing valuable insights for the clinical application of OBP-301 against bone and soft-tissue tumors.

## Supporting information

S1 Table Characteristics and experimental data of clinical tumor specimens. (XLSX)
